# Chinese 1 strain of *Toxoplasma gondii* excreted–secreted antigens negatively modulate Foxp3 *via* inhibition of the TGFßRII/Smad2/Smad3/Smad4 pathway

**DOI:** 10.1111/jcmm.13115

**Published:** 2017-03-16

**Authors:** Jinling Chen, Caiqun Huang, Dandan Zhu, Pei Shen, Yinong Duan, Jianxin Wang, Chunzhao Yang, Liting Wu

**Affiliations:** ^1^ Department of Pathogen Biology School of Medicine Nantong University Nantong Jiangsu China; ^2^ Laboratory Medicine Center Affiliated Hospital of Nantong University Nantong Jiangsu China

**Keywords:** Chinese 1 strain of *Toxoplasma gondii*, excreted–secreted antigens, Foxp3, TGFßRII/Smad2/Smad3/Smad4

## Abstract

*Toxoplasma gondii* is an opportunistic intracellular parasite and is considered an important aetiological factor in the process of abortion, especially as occurs in early gestation. Chinese 1 strain of *T. gondii* is a dominant genotype prevalent in China. Although it is known that early foetal resorption triggered by RH strain of *T. gondii* is attributable to immune mechanisms rather than its direct effect in uterus, the underlying mechanism of the abortion caused by Chinese 1 strain remains unclear. This study was designed to investigate the effect of excreted–secreted antigens (ESA) of Chinese 1 strain of *T. gondii* on the expression of forkhead box transcription factor (Foxp3) as it pertains to early pregnancy and abortion. ESA caused a marked inhibition in the expression of Foxp3 both *in vivo* and *in vitro*. In addition, ESA negatively modulated Smad2 and Smad3 at the posttranslational level. Smad2 siRNA cooperated with ESA to further suppress the level of Foxp3. This inhibitory effect on Foxp3 expression was partially abrogated by overexpression of Smad2, Smad3 and Smad4. Additionally, ESA attenuated the expression of TGFßRII, whereas TGFßRII agonist could profoundly reversed the decreased Foxp3 triggered by ESA. Collectively, the findings suggested that ESA restricted Foxp3 expression by inhibiting TGFßRII/Smad2/Smad3/Smad4 signalling, ultimately resulting in abortion.

## Introduction

*Toxoplasma gondii*, an opportunistic parasite, results in a globally prevalent disease 1. In individuals with normal immune systems, it does not cause serious disease; however, in those with compromised immunity, it can cause toxoplasmic pneumonia and encephalitis 2. Normally, the maternal immune system is considered a state of immunological tolerance, which tolerates the semi‐allogeneic foetus without mounting an immunological rejection. During the early stage of pregnancy, maternal infection with *T. gondii* can lead to such serious outcome as miscarriage, spontaneous abortion or foetal teratogenesis 3. It has long been proposed that the early foetal resorption that coincides with toxoplasmosis is attributable to other mechanisms rather than the direct effect of *T. gondii* proliferation in the uterus 4. In our previous study, we have found that ESA of RH strain *T. gondii* resulted in a decrease in the number and the suppressive capacity of regulatory T cells (Tregs), which could ultimately lead to foetal loss. Although Chinese 1 strain of *T. gondii* is a dominant genotype prevalent in China, the role of ESA on Tregs remains less clear.

It has been defined that Tregs have a major effect on maintaining peripheral tolerance and preventing autoimmune actions as well as tolerating allogeneic organ grafts 5. Furthermore, during pregnancy, Tregs are key players in the tolerance toward the foetus bearing alloantigen 6. A link between the decreased number and the diminished suppressive capacity of Tregs has been linked to immunological rejection of the foetus, an effect that was partially abrogated by adoptive transfer of Tregs from normal pregnant mice into abortion‐prone animals 7. Foxp3 works as a master switch in modulating the development and function of Tregs, and it has been proposed that diminished expression of Foxp3 in endometrial tissue might be responsible for unexplained infertility 8. In our study, we have also found that ESA could negatively moderate Foxp3 in pregnant mice, and hence, speculate that ESA inhibits the function of Tregs *via* suppression of Foxp3, ultimately resulting in abortion.

TGF‐β plays a critical role in the regulation of Foxp3 gene expression 9. The signalling of TGF‐β is initiated by binding of TGF‐β ligands to TGF‐β type II receptor (TβRII) 10. Once bound to TGF‐β, TβRII recruits and stimulates TGF‐β type I receptor (TβRI) protein kinase activity. Then, once activated, TβRI phosphorylates Smad2 and Smad3, allowing them to bind to Smad4. These formed Smad complexes translocate into the nucleus and control Foxp3 expression. In addition, lack of Smad3 resulted in diminished Foxp3 expression triggered by TGF‐β *in vitro* 11. Smad4 deficiency also attenuated *in vitro* TGF‐β‐triggered polarization of Foxp3^+^CD4^+^ T cells 12. This reduction in Foxp3 expression triggered by ESA is likely attributable to the suppression of the TβRII/Smad‐mediated signalling pathway. Therefore, we set out to investigate the effects of ESA on the expression of Foxp3 *in vivo* and *in vitro*, and the mechanism under which these events occur.

## Materials and methods

### Ethics statement

All procedures performed on animals within this study were conducted following the Institutional Animal Care and Use Committee (protocol # NTS‐13‐056) at Nantong University, Jiangsu province. All animal experiments were performed in strict accordance with guidelines from the Regulations for the Administration of Affairs Concerning Experimental Animals (1988.11.1), and efforts were exerted to minimize the suffering of the animals.

### Preparation of parasite antigens

Chinese 1 strain of *T. gondii* establishes and maintains according to our previous study 13. ESA was preformed as previously described 14. To remove endotoxin, ESA was treated with AffinityPak Detoxi‐Gel Endotoxin Removing Gel (Thermo, Fairlawn, OH, USA). A Limulus Amebocyte Lysate assay kit (Lonza, Basel, Switzerland) was used to confirm the removal of endotoxins from the ESA.

### Mice and mating

All experiments were approved by the Institutional Animal Experimental Ethics Committee of Nantong University. Mating of mice was performed as previously described 3. Pregnant mice at gestational day 5 (G5) were injected with ESA (0.05 mg/ml, dissolved in PBS), PBS, RU486 (0.8 mg/ml; Sigma‐Aldrich, St. Louis, MO, USA), respectively. The percentage of abortion was calculated as described previously 15, 16.

### Haematoxylin–eosin staining

Immediately following the euthanasia of pregnant mice, placenta specimens were fixed in 4% (w/v) paraformaldehyde in PBS and dehydrated in a graded sucrose series. Eight micrometer‐thick cryosections of placenta tissue were obtained using a Leica CM1950 Cryostat (Leica, Wetzlar, Germany) and were stained with haematoxylin–eosin. Images from five random fields of the stained placenta cryosections from each mouse were taken with a Leica DM 5000 B microscope (Leica).

### Flow cytometric analysis

Spleens were collected to prepare single‐cell suspensions according to method described in Tang *et al*. 17. For analysis of CD4^+^CD25^+^Foxp3^+^ T cells, a mouse regulatory T‐cell staining kit was used, according to the manufacturer's instructions (eBioscience, San Diego, CA, USA).

### Isolation of Tregs and cell culture

CD4^+^CD25^+^ T cells were isolated from the splenocytes as described previously 7. The purity of the preparations was between 96% and 98% in all experiments. ESA (10 μg/ml) or non‐antigen (Ag)‐specific stimulant ovalbumin (OVA 10 μg/ml) was added to the EL4 cells (the cell resource centre of the Shanghai Institute of Life Science) and incubated for 12 hrs in the presence or absence of TGF‐β1 (5 ng/ml), CD3 (145‐2C11, 1 μg/ml) and CD28 (37.51, 1 μg/ml). Isolated Tregs from normal pregnant mice were exposed to ESA or OVA for 12 hrs.

### Proliferation assay

CD4^+^CD25^−^ T cells purified from control mice were stimulated with 1 μg/ml anti‐CD3 mAb in the presence of CD4^+^CD25^+^ T cells isolated from PBS‐injected, ESA‐injected or RU486‐injected pregnant mice and cultured for 72 hrs. Proliferation was measured with an ELISA 5‐bromo‐2‐deoxyuridine (BrdU) kit (Roche Applied Science, Mannheim, Germany), according to the manufacturer's instructions 18.

### Real‐time quantitative PCR

For real‐time quantitative PCR analysis, total RNA was isolated from purified CD4^+^CD25^+^ T cells using Trizol reagent (Invitrogen, San Diego, CA, USA), and primer sequences used were as follows: b‐actin, forward: GCTCTGGCTCCT AGCACCAT; reverse: GATCCACACAGAGTACTTGCGC. Foxp3, forward: GGCCCTTCTCCAGGACAGA; reverse: GCTGATCATGGCTGGGTTGT 7. Smad4, forward: ACACCAACAAGTAACGATGCC; reverse: GCAAAGGTTTCACTTTCCCCA. TβRII, forward: CCGCTGCATATCGTCCTGTG; reverse: AGTGGATGGATGGTCCTATTACA.

### Western blot analysis

Western blot was performed as previously described 3. TβRII and Smad4 (Santa Cruz Biotechnology Santa Cruz, CA, USA, USA), P‐Smad2, P‐Smad3, Smad2, Smad3 (Cell Signaling Technology, Danvers, MA, USA) and Foxp3 (Abcam, Cambridge, MA, USA) were used for the detection of proteins. The signals were visualized by enhanced chemiluminescence (ECL; Merck, Darmstadt, Germany). Quantification analysis was performed using the GeneTools software from Syngene (Cambridge, UK).

### Immunofluorescence analysis

Immunofluorescence was performed as described previously 19. Briefly, slides with cells incubated with anti‐phospho‐Smad3 and Foxp3 overnight at 4°C after blockage. The immune reactivity was visualized by incubating the slides with a donkey–anti‐rabbit IgG antibody conjugated with Alexa Fluor 568 (Invitrogen) and Hoechst 33342. The fluorescent intensity for at least six regions per section was measured using ImageJ software (National Institutes of Health, Bethesda, MD, USA).

### RNA interference

Small interfering RNAs (siRNAs) were designed by Genepharma (Shanghai, China). The sequence of siRNAs corresponding to mouse Smad2 is located within exon 5: 5′‐GCUGAACUGUCUCCUACUATT‐3′. A universal control siRNA was used as a nonspecific control. EL4 cells were transfected with siRNA duplex using Lipofectamine 2000 (Invitrogen), according to manufacturer's instructions.

### Cell electroporation

The Smad2, Smad3 and Smad4 genes were amplified and cloned into a pcDNA3.1 vector (Novagen, Madison, WI, USA) using the following primer pairs: Smad2, sense primer: 5′‐AAGCTTGCCACCATGTCGTCCATCTTGCCATTC‐3′; antisense primer: 5′‐GAATTCTTTGCTCTGGAATTTTTGGATAG‐3′, Smad3, sense primer: 5′‐ GAAT TCGCCACCATGTCGTCCATCCTGCCCTTC‐3′; antisense primer: 5′‐ CTCGAGC CTGGGGTTTTCTTCTGTGGTC‐3′, Smad4, sense primer: 5′‐GAATTCGCCACCAT GGACAATATGTCTATAACAAATACAC‐3′; antisense primer: 5′‐CTCGAGTCAGT CTAAAGGCTGTGGGTC‐3′. Transfection of EL4 cells was achieved using the Amaxa Nucleofector system. A total of 5 × 10^6^ EL4 cells were placed in 100 μl electroporation solution (Entranster‐E) with 2 μg pcDNA3.1‐Smad2, pcDNA3.1‐Smad3 or pcDNA3.1‐Smad4, and transfected using the Nucleofector program.

### ELISA

EL4 cells were plated into 12‐well plates at a density of 1 × 10^6^ cells/well. ESA (10 μg/ml) or OVA (10 μg/ml) was added to the EL4 cells and incubated for 12 hrs in the presence or absence of TGF‐β1 (5 ng/ml). The supernatants of EL4s were collected treated for 12 hrs. Mouse TGF‐β1 concentration in the supernatants was quantitatively measured using a commercial ELISA kit (Boster, Wuhan, China) following the manufacturer's instructions. ELISA experiments were repeated at least three times.

### Statistical analysis

The statistical significance of differences in the means of experimental groups was determined using Prism software (GraphPad, San Diego, CA). Data from two groups were analysed for statistical significance with Student's *t*‐test. Multiple comparisons were made with one‐way anova.

## Results

### The reduced frequency and function of CD4^+^CD25^+^Foxp3^+^ T cells contributed to the abortion induced by *T. gondii* ESA or RU486

**Figure 1:**
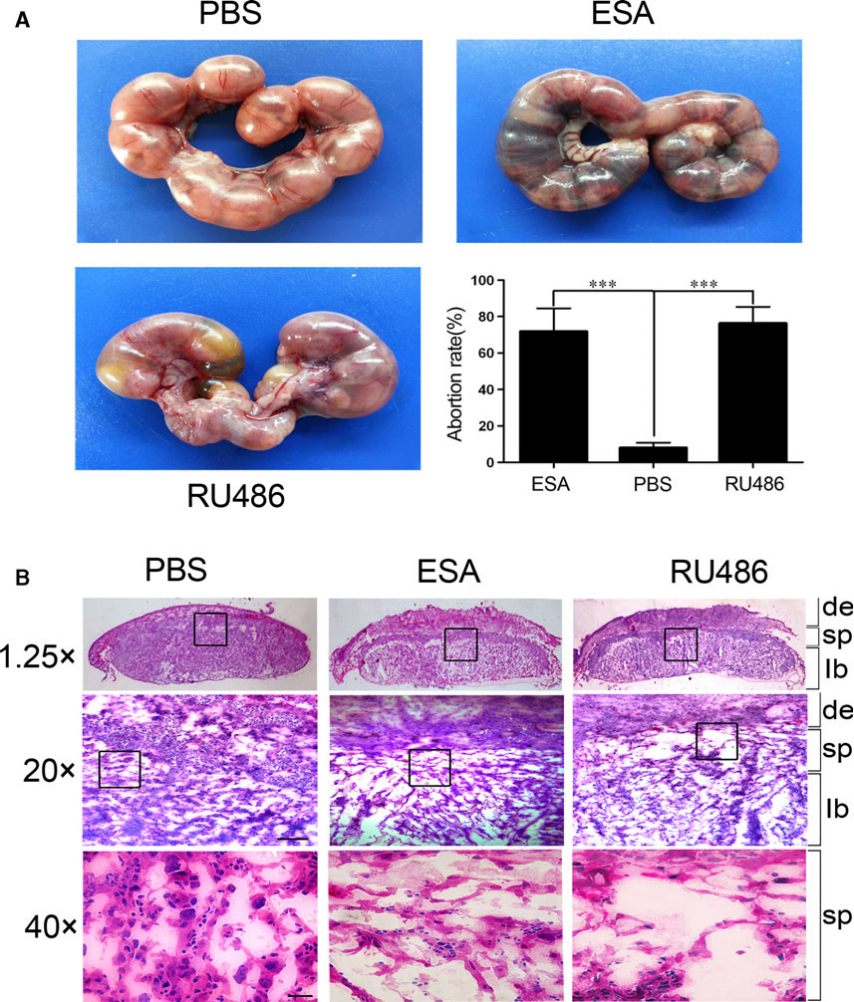
*Toxoplasma gondii* ESA or RU486 results in the abortion. (**A**) Representative pictures of uteri from mice injected with ESA, PBS and RU486 at G5. All animals were killed at G18. The abortion rate was calculated as the ratio of abortion sites to the total numbers of implantation sites. Statistical differences between groups are shown as follows: ***P < 0.001. (**B**) Placental anomalies in the labyrinth of embryos. Schematic representation of the mouse placenta showing the labyrinth (lb), spongiotrophoblast (sp) and the maternal decidua (de).

*Toxoplasma gondii* infection triggers early embryonic death and resorption, foetal death, abortion and stillbirth 20. It has been recognized that this phenomenon is not solely due to the direct effect of its proliferation but that other mechanisms may be largely responsible 21. ESA, which represents the majority of the *T. gondii* circulating antigens in sera from hosts with acute infection 22, could be found at both its encysted bradyzoite and tachyzoite stages 23. Pregnant mice at G5 were injected with ESA, PBS and RU486 intraperitoneally. At euthanasia at G18, nearly all of the embryos and placentas exhibited a necrotic and haemorrhagic appearance following the administration of ESA. RU486 injection resulted in visible foetal abnormality, similarly to that observed in the mice in the ESA group (Fig. 1A). To further characterize the labyrinth defects in the placenta, we examined the fine structure of the interface between the maternal and foetal blood compartments (Fig. 1B) in G18 placenta. As can be seen in Figure 1B, the placental labyrinth showed the typical interhaemal barrier separating foetal blood vessels and maternal lacunae.

**Figure 2:**
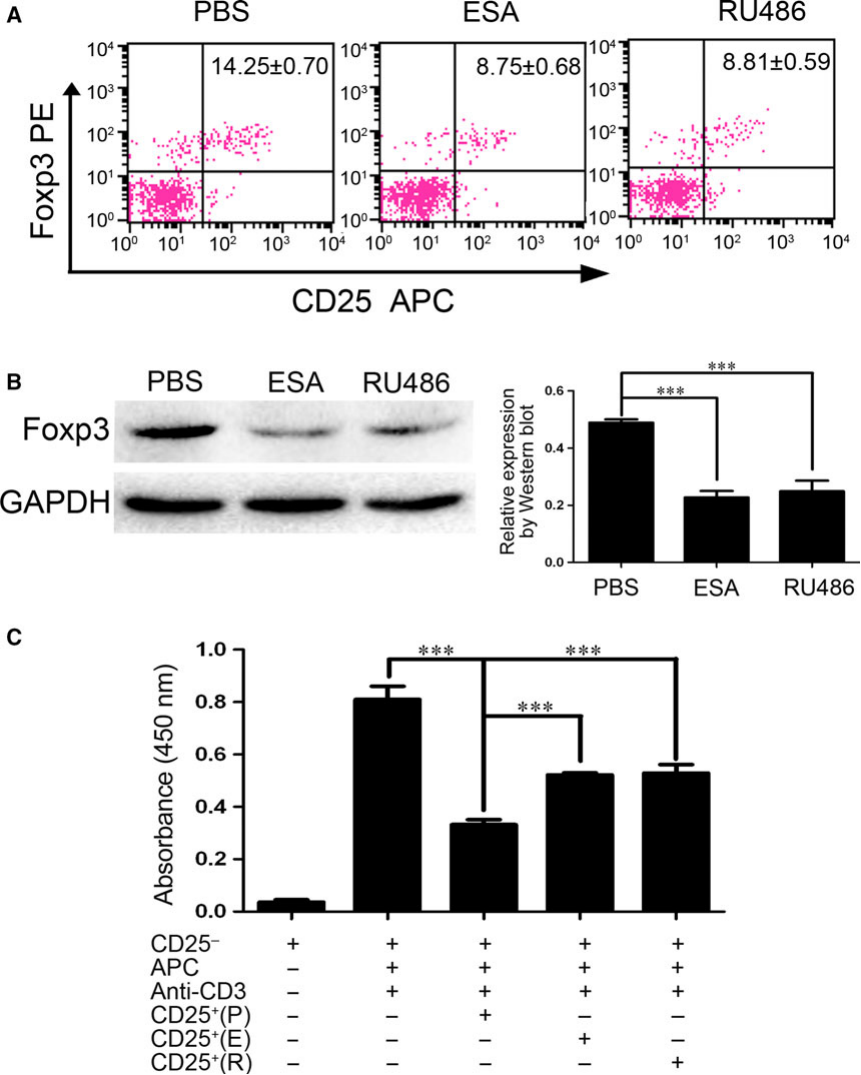
The reduced frequency and function of CD4^+^CD25^+^Foxp3^+^T cells contribute to the abortion induced by ESA or RU486. (**A**) Percentages of CD4^+^CD25^+^Foxp3^+^ T cells from spleens analysed by flow cytometry, gated on CD4^+^ cells. (**B**) Foxp3 protein from placenta was analysed by Western blot as indicated. One representative result is shown from three independent experiments performed. ***P < 0.001. (**C**) Responder CD4^+^CD25^−^ T cells (1 × 10^5^/well) from naive mice were cultured with naive, irradiated APC (1 × 10^5^/well) and CD4^+^CD25^+^T cells (5 × 10^4^/well) harvested from pregnant mice injected with PBS (P), RU486 (R) and *Toxoplasma gondii* ESA (E), respectively. Proliferation was measured with an ELISA 5‐bromo‐2‐deoxyuridine (BrdU) kit. The absorbance at 450 nm was measured in an ELISA reader. The data shown were performed in triplicate and were representative of three independent experiments. ***P < 0.001.

Based on the role of CD4^+^CD25^+^Foxp3^+^ T cells in achieving maternal–foetal immunotolerance, the number of CD4^+^CD25^+^Foxp3^+^ T cells in splenocytes was assessed by flow cytometry. The data show that ESA or RU486 did indeed attenuate the frequency of CD4^+^CD25^+^Foxp3^+^ T cells in abortion‐prone mice (Fig. 2A). The regulation of the immune response at the maternal–foetal interface is complex and promotes tolerance of paternally derived antigens 24. We assessed the expression level of Foxp3 protein in the placentas of mice to determine whether the reduction in CD4^+^CD25^+^ Tregs occurred at the maternal–foetal interface. The results showed a decrease in placental Foxp3, consistent with global changes seen in CD4^+^CD25^+^Foxp3^+^ T cells (Fig. 2B). To assess the function of CD4^+^CD25^+^Foxp3^+^ T cells, primary Treg cells were isolated from the mice. Administration of ESA or RU486 resulted in the attenuation of the inhibitory capacity of CD4^+^CD25^+^ T cells (Fig. 2C), which suggested that the abortion triggered by ESA or RU486 was attributable to the reduced frequency and function of CD4^+^CD25^+^Foxp3^+^ T cells.

### The reduced expression of Foxp3 induced by ESA is related to Smad2/3/4 signalling pathway

**Figure 3:**
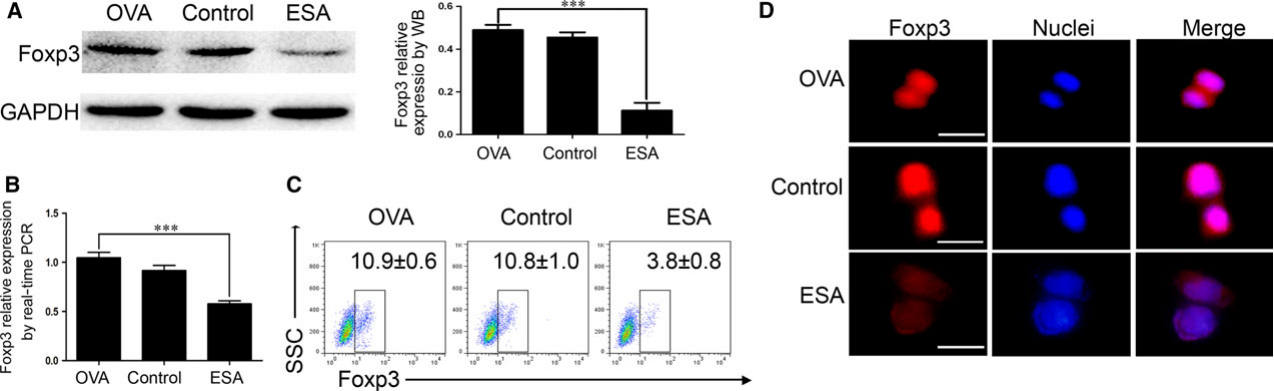
ESA reduced the expression of Foxp3. (**A**) The activity of Foxp3 was significantly decreased in the EL4 cells treated with ESA (10 μg/ml for 12 hrs), compared to the control group or OVA group. ***P < 0.01. (**B**) The expression of Foxp3 was diminished in the primary Tregs measured by real‐time PCR, compared to the control group or OVA group. ***P < 0.01. (**C**) Percentages of Foxp3^+^ cells in the EL4 cells analysed by flow cytometry. (**D**) The images were photographed under fluorescence microscopy. The nuclei were stained by Hoechst 33342. Bar: 20 μm.

**Figure 4:**
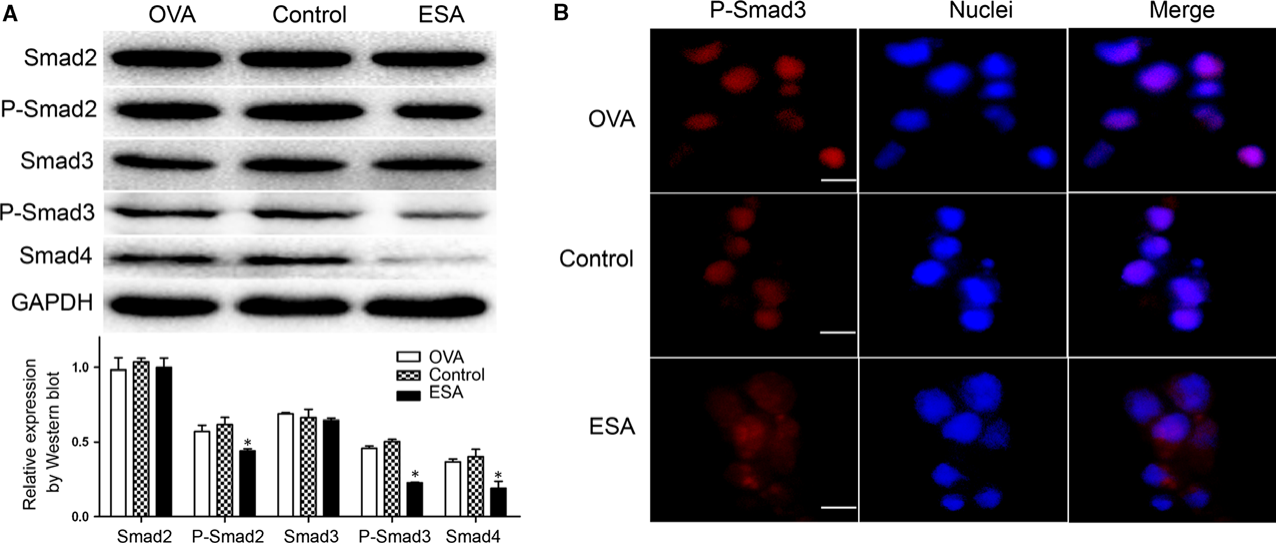
ESA inhibited the expression of P‐Smad2, P‐Smad3 and Smad4. (**A**) The activity of P‐Smad2, P‐Smad3 and Smad4 significantly was decreased, while the expression of Smad2 and Smad3 unaffected in the EL4 cells treated with ESA (10 μg/ml for 12 hrs) compared to the control group or OVA group. *P < 0.05. (**B**) The images were photographed under fluorescence microscopy. The nuclei were stained by Hoechst 33342. The level of P‐Smad3 was down‐regulated in the cells after being exposed to ESA compared to control group. Bar: 20 μm.

Foxp3 is a major transcription factor for the development and function of regulatory T cells. Foxp3 has been implicated in immunoregulation, autoimmune diseases, infections and tumour immune evasion/escape 25. To gauge the effects of ESA on the expression of Foxp3, we used the EL4 cell line, which expresses abundant Foxp3 only upon stimulation and maintains many T‐cell properties 26. After EL4 cells were exposed to ESA for 12 hrs, Foxp3 was significantly diminished (Fig. 3A). To further confirm the role of ESA on the primary Tregs, isolated Tregs were exposed to ESA, which exhibited an inhibitory effect on the expression of Foxp3 at the mRNA level (Fig. 3B). Foxp3 expression was analysed in EL4 cells by flow cytometry. EL4 cells displayed the markedly decrease in the expression of Foxp3 after the exposure of ESA (Fig 3C). Moreover, the cells in the ESA group, not in control group, showed much lower fluorescence intensity of Foxp3 (Fig. 3D). Thus, it was evident that ESA markedly suppressed the expression of Foxp3. The Smad signalling pathway plays an essential role in the process of inducing and maintaining Foxp3 expression 27. To establish a link between Smad signalling pathway and decreased Foxp3 triggered by ESA, we tested the levels of Smad2/3 and Smad4. We found that ESA inhibited Smad4 and phosphorylated Smad2/3, not the total Smad2/3 (Fig. 4A). We further observed nuclear translocation of phosphorylated Smad3. The cells in the ESA group showed much lower fluorescence intensity of P‐Smad3 inside the nuclei (Fig. 4B). This suggested that ESA suppressed the expression of Foxp3 *via* the attenuation of the phosphorylation of Smad2/3 and Smad4.

### The ESA‐induced downregulation of Foxp3 in EL4 cells was linked with P‐Smad2

**Figure 5:**
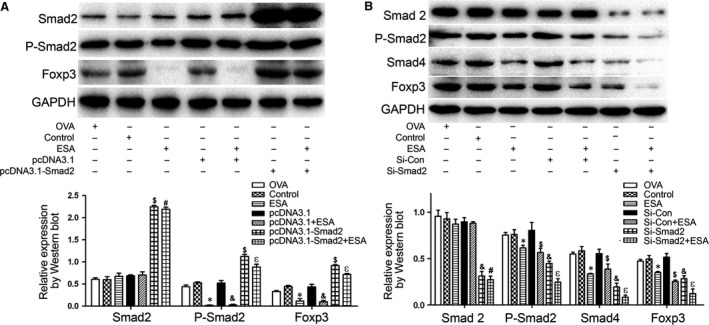
The decreased expression of Foxp3 induced by ESA was associated with P‐Smad2. (**A**) Cells were transfected with pcDNA3.1‐Smad2 or pcDNA3.1 vector with or without ESA. The expression levels of Smad2, P‐Smad2 and Foxp3 were detected by Western blot. *P < 0.05 compared to the control group. &*P* < 0.05 compared to pcDNA3.1 vector group. $*P* < 0.05 compared to pcDNA3.1 vector group. εp < 0.05 compared to pcDNA3.1‐Smad2 group. #*P* > 0.05 compared to pcDNA3.1‐Smad2 group. (**B**) The EL4 cells were transfected with Smad2‐target siRNA or control siRNA with or without ESA. *P < 0.05 compared to the control group. $*P* < 0.05 compared to Si‐Con group. &*P* < 0.05 compared to Si‐Con group. εP < 0.05 compared to compared to Si‐Smad2 group. #*P* > 0.05 compared to Si‐Smad2 group.

Smad2 and Smad3 are key regulators for TGF‐β‐mediated Tregs. Phosphorylation of Smad2/3 primarily mediates TGF‐β1‐induced transcriptional regulation and translocates into the nucleus to regulate genes 28. The requirement of Smad2 for ESA‐mediated Foxp3 regulation was further verified by interference and over expression of Smad2 in EL4 cells. Transfection of pcDNA3.1‐Smad2 into EL4 cells resulted in an obvious enhancement in the expression of Smad2, P‐Smad2 and Foxp3 (Fig. 5A). To further explore the molecular pathways underlying the role of Smad2 on decreased Foxp3, we used siRNA designed specifically against Smad2 to test the expression of downstream target proteins. Our results revealed that knockdown of Smad2 could inhibit Smad4 and Foxp3 (Fig. 5B). Furthermore, ESA assisted Smad2 siRNAs to further suppress the level of P‐Smad2, Smad4, and Foxp3. Together, these results indicate that ESA induces downregulation of Foxp3 in EL4 cells *via* downregulation of P‐Smad2.

### ESA induces downregulation of Foxp3 in EL4 cells *via* Smad3/Smad4

**Figure 6:**
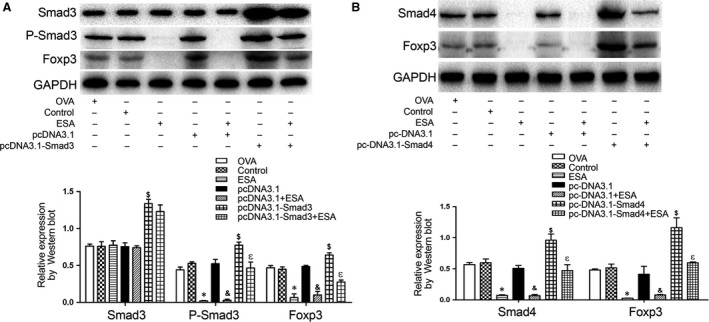
ESA‐induced downregulation of Foxp3 in EL4 cells *via* Smad3/Smad4. (**A**) Cells were transfected with pcDNA3.1‐Smad3 or pcDNA3.1 vector with or without ESA. The expression levels of Smad3, P‐Smad3 and Foxp3 were detected by Western blot. *P < 0.05 compared to the control group. &*P* < 0.05 compared to pcDNA3.1 vector group. $*P* < 0.05 compared to pcDNA3.1 vector group. εP < 0.05 compared to pcDNA3.1‐Smad3 vector group. (**B**) Cells were transfected with pcDNA3.1‐Smad4 or pcDNA3.1 vector with or without ESA. The expression levels of Smad4 and Foxp3 were detected by Western blot. *P < 0.05 compared to the control group. &*P* < 0.05 compared to pcDNA3.1 vector group. $*P* < 0.05 compared to pcDNA3.1 vector group. εP < 0.05 compared to the pcDNA3.1‐Smad4 group.

A recent report suggested that Smad3 regulates Foxp3 enhancer activity and that induction of Foxp3 expression is hindered by the Smad3 inhibitor SIS3 26. To be certain of the requirement of Smad3 for ESA‐mediated Foxp3 regulation, we transferred pcDNA3.1‐Smad3 into EL4 cells and found that expression of Smad3 was enhanced double that seen in the control group (Fig. 6A). Simultaneously, there was an obvious improvement in the levels of P‐Smad3 and Foxp3 with pcDNA3.1‐Smad3. Nevertheless, the increased expression of Smad4 and Foxp3 induced by pcDNA3.1‐Smad4 was inhibited by ESA exposure (Fig. 6B). These data suggested that the ESA‐induced downregulation of Foxp3 in EL4 cells occurred *via* Smad3/Smad4.

### Downregulation of ESA‐induced Foxp3 expression *via* the inhibition of TGFßRII

**Figure 7:**
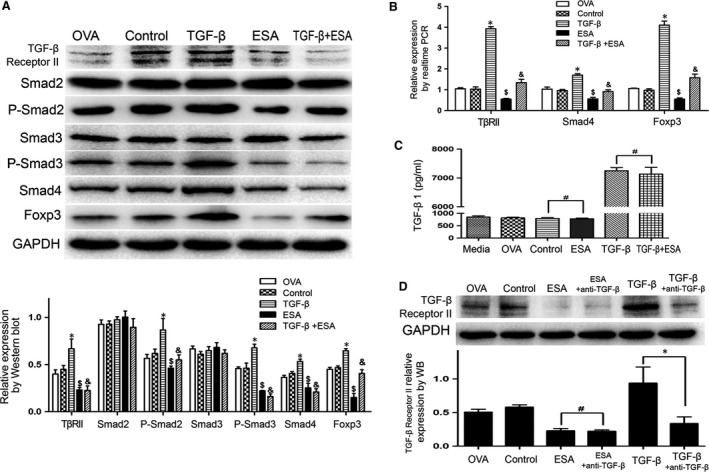
Downregulation of ESA‐induced Foxp3 expression *via* the inhibition of TβRII. (**A**) The EL4 cells were treated with or without ESA and TβRII agonist (TGF‐β1). The expression of TßRII, Smad2, P‐Smad2, Smad3, P‐Smad3, Smad4 and Foxp3 was analysed by Western blot. The increased expression of TßRII, P‐Smad2, P‐Smad3, Smad4 and Foxp3 induced by TGF‐β1 could all be reversed with ESA supplementation. *P < 0.05 compared to the control group. $*P* < 0.05 compared to the control group. &*P* < 0.05 compared to the TGF‐β1 group. (**B**) The relative expression of TßRII, Smad4 and Foxp3 in the primary Tregs measured by real‐time PCR. *P < 0.05 compared to the control group. $*P* < 0.05 compared to the control group. &*P* < 0.05 compared to the TGF‐β1 group. (**C**) The level of TGF‐β1 measured by ELISA. #*P* > 0.05. (**D**) The expression of TßRII were analysed by Western blot. #*P* > 0.05. *P < 0.05.

TGF‐β facilitates Foxp3 induction through TβRI and TβRII. The phosphorylation and activation of TβRI could be mediated by TGF‐β binding to TβRII. Through interaction with TβRI, the phosphorylated Smad2/3 complex dissociates to form a heterotrimeric complex with Smad4, an event which is then followed by their translocation into the nucleus to further activate Foxp3 29, 30. As suggested, TβRII is viewed as a key switch in the TGF‐β signalling pathway. Our study demonstrated that ESA could inhibit the expression of TβRII (Fig. 7A). To further confirm the TβRII effect on the decreased Foxp3 mediated by ESA, the TβRII agonist (TGF‐β) was exposed to EL4 cells. The reduced P‐Smad2/Smad3 and Foxp3 were reversed by TβRII agonist (Fig. 7A). To confirm that ESA exhibited an inhibitory effect on the expression of Smad4 and TβRII in the primary Tregs, Smad4 and TβRII expression in the primary Tregs were measured by real‐time PCR. Smad4 and TβRII expression at the mRNA level were inhibited by ESA (Fig. 7B). To rule out the possibility that ESA might influence the availability of TGF‐ß, rather than influencing the signalling pathway, the level of TGF‐ß was assayed by ELISA. No significant difference was observed in the supernatant between either the ESA or OVA groups (Fig. 7C), suggesting that ESA has no effect on the level of TGF‐ß. Then, anti‐TGF‐ß was used to down‐regulate TGF‐ß. If ESA displays the inhibitory effect on the TβRII expression by influencing the availability of TGF‐ß, ESA fails to inhibit the TβRII expression with anti‐TGF‐ß supplementation. However, ESA still can markedly diminish TβRII expression, suggesting that ESA has no effect on the availability of TGF‐ß (Fig. 7D). Collectively, these results indicated that ESA‐inhibited Foxp3 was mediated by inactivation of TβRII.

## Discussion

Infection with *T. gondii*, an opportunistic intracellular parasite, potentially results in miscarriage, spontaneous abortion or foetal teratogenesis. Substantial evidence indicates that early foetal teratogenesis is independent of vertical infection 7, 21. The diminished number and function of Tregs triggered by *T. gondii* were responsible for pregnancy failure 4. Chinese 1 strain of *T. gondii* is predominantly prevalent in China 31. Some researchers demonstrated that maternal infection with *T. gondii* ended in pregnancy failure, due to trophoblast apoptosis 32. Nevertheless, the effect of *T. gondii* antigen on the abortion remains unclear. Here we show that dysfunction of Tregs induced by *T. gondii* antigen (ESA) leads to the adverse outcome of pregnancy. RU486 (Mifepristone)‐induced abortion in mice is attributable to the inhibition of the number and the function of Tregs (Fig. 2). The data suggested Tregs were a key regulator in the abortion induced by *T. gondii* ESA or RU486.

Foxp3, a key transcription factor for Tregs, is the critical regulatory gene in the development and function of Tregs 33, 34. Foxp3‐deficient recombinant mice suppresses the regulatory function of Treg cells 35. In line with our findings, Li X revealed that RU486‐induced abortion was partially due to the reduced Foxp3 36. Cimetidine resulted in the downregulation of Foxp3 *via* E3 ligase Stub1‐mediated proteasomal degradation 37. We found that EL4 cells have shown a decrease in Foxp3 protein upon exposure to ESA. Nevertheless, prior research indicates that lipopolysaccharides (LPS) or inflammatory cytokines can negatively affect Foxp3 protein stability at the post‐translational level through the E3 ubiquitin ligase activity of Stub1 38, a finding that reflects the role of LPS on the negative regulation of Foxp3 expression. We thereby removed LPS from ESA to rule out the possibility that any present endotoxins contributed to the degradation of Foxp3. Upon removal of endotoxin, ESA still negatively modulate the expression of Foxp3.

Although research has demonstrated that ESA results in the loss of Foxp3 expression *in vivo* and *in vitro*, the underlying mechanism remains unclear. Smad2 and Smad3 contribute to TGF‐β‐mediated Foxp3 induction, and Smad3 and NFAT cooperate to induce Foxp3 expression through its enhancer 26. Lack of Smad3 diminishes the Foxp3 expression triggered by TGF‐β *in vitro* 11. All‐trans‐retinoic acid (ATRA) likely promoted the induction of Foxp3^+^ cells through the early initiation of Smad2/3 activation 39. During the cell conversion of induced Tregs (iTregs), Smad2 and Smad3 are key regulators for the activation of the Foxp3 expression *via* direct binding to a Foxp3 CNS1 40. Xiao *et al*. showed that ATRA facilitated the differentiation of iTregs by increasing Smad3 expression and phosphorylation 41. In our study, ESA stimulation markedly suppressed the expression of Foxp3 through inhibiting the phosphorylation of Smad2 and Smad3, while the overexpression of Smad2 and Smad3 abrogated the ESA‐triggered downregulation of Foxp3.

Smad4 is defined as an important universal coregulator of TGF‐β signalling. Nevertheless, significant differences between T‐cell‐specific Smad4^−/−^ mice and Smad2^−/−^ Smad3^−/−^ mice were observed. TGF‐β‐triggered Foxp3 induction in T cells is partially reversed with knockdown of Smad4, but is eliminated entirely in CD4^+^ T cells from Smad2 and Smad3 double knockout mice 42. In our study, overexpression of Smad4 could abrogate ESA‐mediated Foxp3 downregulation. Phosphorylated Smad2 and Smad3, as well as the receptor‐regulated Smads (R‐Smads), form a trimer with Smad 4, which translocates into the nucleus. Then, the cooperation between the complex of Smad2/3‐Smad4 and DNA binding transcription factors mediates the gene expression of Foxp3 11. The decreased expression of Foxp3 triggered by ESA is likely due to the reduced level of phosphorylated Smad 2 and Smad 3, and Smad 4.

In the presence of antigen stimulation, TGF‐β converts CD4^+^ T cells to Tregs and induces Foxp3 expression, primarily through TβRI and TβRII 5. Some studies indicated that Melittin inhibits TGF‐β‐induced pro‐fibrotic gene expression *via* the suppression of the TβRII‐Smad, ERK1/2 and the JNK‐mediated signalling pathway 29. In line with our findings, ESA elicited an inhibitory effect on the expression of TβRII. Meanwhile, TβRII agonist could abrogate the ESA‐induced reduction in Foxp3 expression (Fig. 7A). Thus, ESA can inhibit TβRII‐mediated activation of Smad2/Smad3/Smad4 signalling, ultimately resulting in the loss of Foxp3.

**Figure 8:**
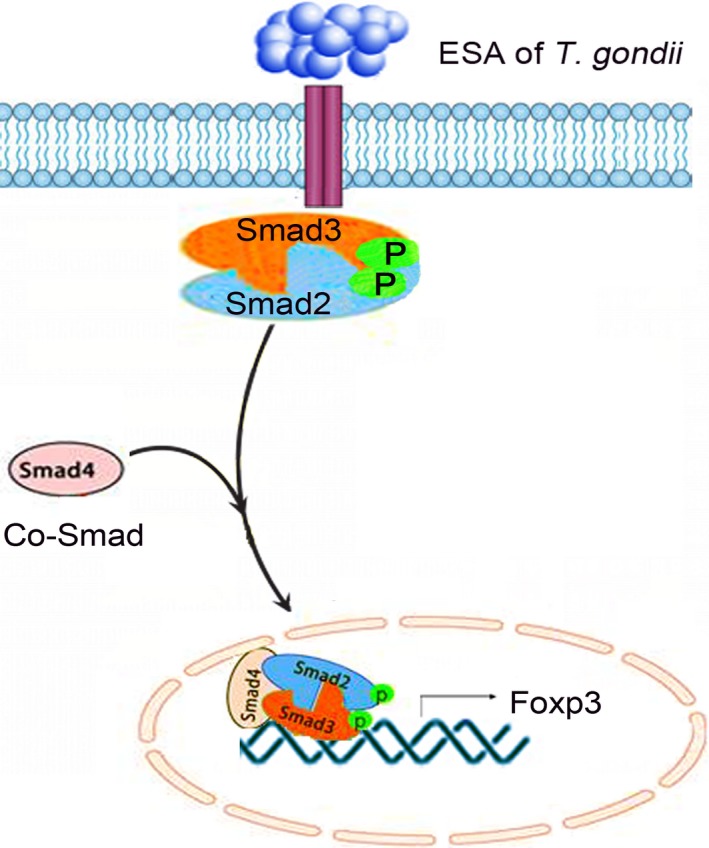
Proposed mechanism of ESA‐mediated downregulation of Foxp3 in regulatory T cells. ESA down‐regulates the expression of P‐Smad2/Smad3 *via* the inactivation of TßRII. Then, it may contribute to the down regulation of Smad4, leading to the diminished Foxp3. Ultimately, the reduced Foxp3 might be responsible for the abortion caused by ESA.

In summary, our findings provide evidence supporting a possible mechanism by which Chinese 1 strain of *T. gondii* ESA induces abortion in mice. In general, we believe it is likely that ESA inhibits the phosphorylation of Smad 2 and Smad 3, as well as Smad4, ultimately restricting Foxp3 expression (Fig. 8).

## Conflict of interest

The authors declare that they have no conflict of interest.
